# Quantitative trait loci analysis for molecular markers linked to agricultural traits of *Pleurotus ostreatus*

**DOI:** 10.1371/journal.pone.0308832

**Published:** 2024-08-12

**Authors:** Jae-San Ryu, Bokyung Park, Arend F. van Peer, Kyeong Sook Na, Song Hee Lee

**Affiliations:** 1 Department of Mushroom Science, Korea National University of Agriculture and Fisheries, Jeonju, Jeonju-si, Jeollabuk-do, South Korea; 2 Gyeongsangnam-do Agricultural Research and Extension Services, Jinju, Republic of Korea; 3 Plant Breeding, Wageningen University and Research, PB Wageningen, The Netherlands; 4 Plant Immunity Research Center, Seoul National University, Seoul, Republic of Korea; VIT University, INDIA

## Abstract

*Pleurotus ostreatus* is a global mushroom crop with nutritional and medicinal benefits. However, the genetic basis of several commercial traits remains unknown. To address this, we analyzed the quantitative trait loci (QTLs) for two representative cultivars, "Heuktari" and "Miso," with apparently distinct alleles. A genetic map with 11 linkage groups was constructed, in which 27 QTLs were assigned to 14 traits. The explained phenotypic variations in QTLs ranged from 7.8% to 22.0%. Relatively high LOD values of 6.190 and 5.485 were estimated for the pinheading period and the number of valid stipes, respectively. Some QTL-derived molecular markers showed potential enhancement rates of selection precision in inbred lines, especially for cap shape (50%) and cap thickness (30%). Candidate genes were inferred from the QTL regions and validated using qRT-PCR, particularly for the cysteine and glutathione pathway, in relation to cap yellowness. The molecular markers in this study are expected to facilitate the breeding of the Heuktari and Miso lines and provide probes to identify related genes in *P*. *ostreatus*.

## Introduction

*Pleurotus ostreatus*, the second-most produced mushroom worldwide [1], is rich in nutritional and therapeutic components, resulting in increasing attention among market players [2]. Global production of oyster mushroom reached 9.3 million tons in 2021 [3] with Korea contributing 96,982 tons in the same year (https://www.mafra.go.kr/). Asia remains the major producer and Europe and North America express increasing interest. As the production of *P*. *ostreatus* increases and the market demands new varieties to meet consumption trends, efficient breeding methods are required to develop these varieties. The preferred mushroom traits vary and are influenced by geographical area, ethnic group, and consumer culture, which intensify their complexity. Breeding new varieties with improved traits is a complicated and time-consuming process. Important traits, such as color, yield, and shape, are often polygenic, and their interactions are unknown [4, 5]. Marker-assisted selection (MAS) based on quantitative trait loci (QTLs) and candidate genes facilitates the selection of progeny with favorable traits at the mycelial stage using markers instead of relying on phenotypes alone [6–8]. QTL analyses have been performed for the genus *Pleurotus* [9–12]. Furthermore, the combination of QTL and genome sequences can be used to identify the corresponding trait genes. In *Pleurotus cornucopiae*, QTLs and corresponding genes conferring cap color have been reported using RNAi [11]. QTLs controlling agronomically important phenotypes in *Pleurotus eryngii* have been previously described [12]. However, several useful traits remain unexplored.

The *P*. *ostreatus* cultivars, Heuktari and Miso, exhibit contrasting phenotypes. Heuktari is characterized by high yield, a dark cap color, convex cap shape, and adaptability to mid-high temperatures, and Miso is characterized by a white cap color, infundibuliform cap shape, and preference for lower temperatures [13, 14]. The popularity of these two cultivars and their contrasting alleles with different phenotypes make them promising lines for breeding and genetic studies.

In this study, we constructed a linkage group and mapped the QTLs in a segregated population derived from Heuktari and Miso cultivars. The genetic map and established QTLs of this study will aid in the creation of selection markers for desirable mushroom traits and the identification of corresponding genes.

## Materials and methods

### Strains and growth conditions

*P*. *ostreatus* Heuktari (KNR2197), Miso (KNR 2097), and the wild-type strain (KNR2247) were obtained from Gyeonggi-Do Agricultural Research and Extension Services and Gyeongsangnam-do Agricultural Research and Extension Services. All strains were cultured in a mushroom complete medium (0.2% peptone, 0.2% yeast extract, 2.0% glucose, 0.05% MgSO_4_·7H_2_O, 0.05% K_2_HPO_4_, and 0.046% KH_2_PO_4_; KisanBio, Seoul, Korea) at 25.0°C in the dark. The strains were maintained fresh by periodic transfer.

### Construction of segregating populations

**Fig 1:**
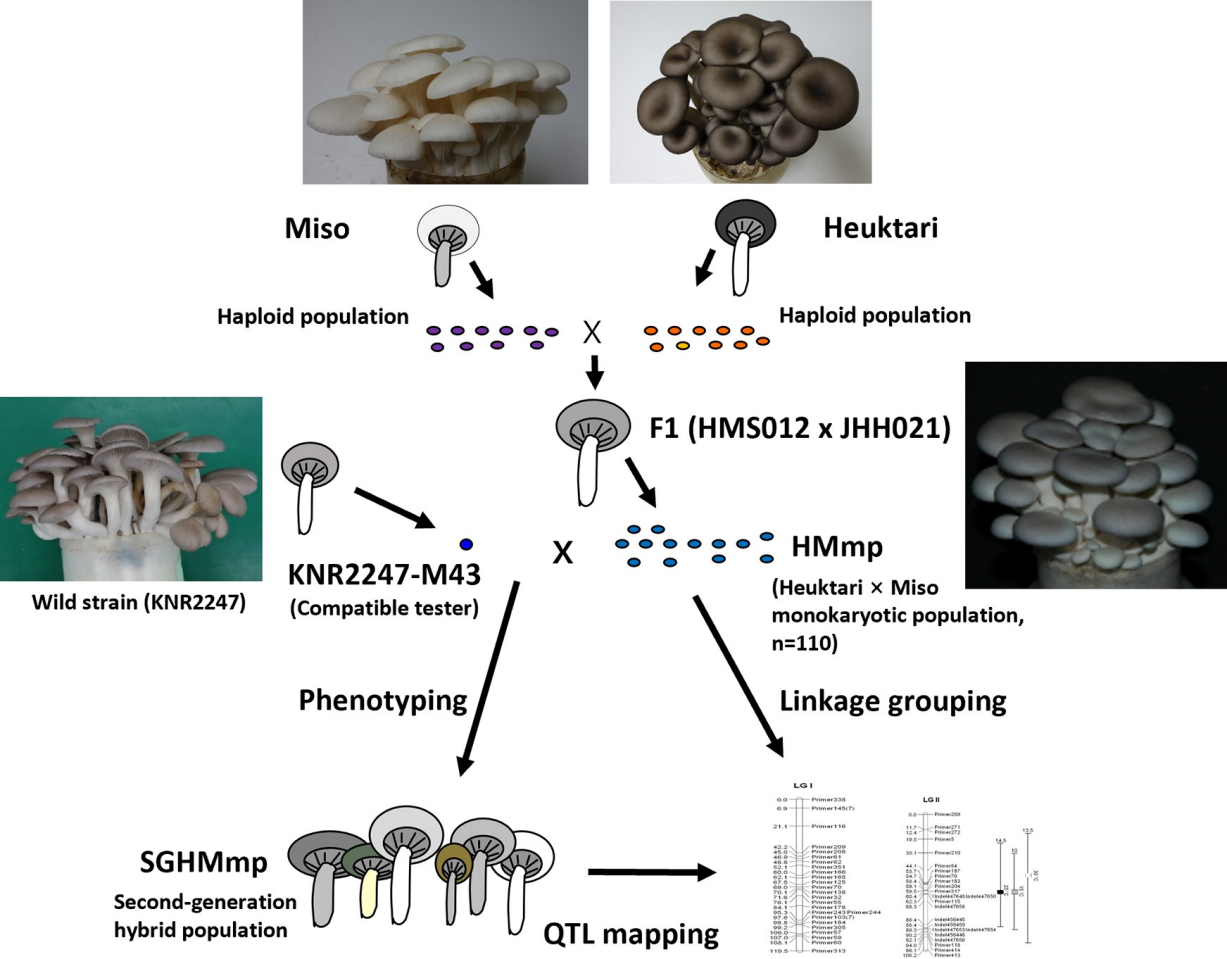
Strategy for QTL analysis in *Pleurotus ostreatus*. Linkage grouping was accomplished through polymorphisms of SSRs in a segregated haploid population (HMmp). Phenotyping was performed in the dikaryotic population (SGHMmp) obtained by mating a compatible tester with a segregated haploid population. QTL analysis was performed using linkage information and traits data.

To obtain F1 hybrids with phenotypic intermediates between Heuktari and Miso (S1 Fig in S1 File), 100 hybrids were generated by crossing 10 Heuktari monokaryons with 10 Miso monokaryons. After the evaluation of phenotypes, HMS012 × JHH021 was selected based on its median values in major traits, cap and stipe lightness, yield, and length (their distribution frequencies are shown in S2 Fig in S1 File). Monokaryons were randomly selected from the HMS012 × JHH021 fruiting bodies to develop an “F2” opulation (HMmp; *n* = 110). The HMmp population was crossed with KNR2247-M43 (a monokaryon of KNR2247) to generate a second-generation hybrid population (SGHMmp) to evaluate the fruiting body phenotypes. KNR2247 exhibited wild-type phenotypes and genetic distances at the A- and B-mating-type loci, making it compatible with all individuals in the HMmp population. The pedigree scheme is illustrated in Fig 1.

### Fructification and phenotypic evaluation

The medium for fruiting body production comprised pine sawdust, rice bran, and cottonseed hulls in a 70:20:10 (v/v) ratio, with a water content of 68% ± 10%. Approximately 770 g ± 10% of the medium was packed into 1,100 mL polypropylene (PP) bottles and then subjected to steam sterilization at 121°C for 90 min. The cooled substrate was inoculated with 15 mL of 10-day-old pre-cultured MCM broth and placed in a dark incubation room at 20°C and 65% RH for 25 days. After incubation, the upper substrate (approximately 10 mm) was scraped off to induce primordium formation. The bottles were then placed upside down in a room maintained at 15°C and 95% RH, with cool white fluorescent light (approximately 150 lux). When the fruiting body reached a height of 1.5 to 2.0 cm, the bottles were turned upright.

Four growing units (bottles) were used to assess most traits, and three representative fruiting bodies were selected from each bottle. Mushrooms were harvested when the cap was approximately 70% open. The periods of pinheading (primordia appearance time from the removal of the old medium) and earliness (harvest time from the removal of the old medium) were measured. The yield was measured by weighing all mushrooms with a valid stipe from each bottle. The number of valid stipes in individual fruiting bodies with a marketable quality of over 3 g was counted. Length was measured from the base of the stipe to the top of cap (full length). Stipe length was measured from the base of the stipe to the inflection point between the gill and the stipe. The shape of the cap was scored using a 5-point scale (1 = infundibuliform to 5 = convex). For example, the cap of Heuktari was scored as 1, and Miso was scored as 3 (S1 Fig in S1 File). The thickness of the cap was measured between the surface of the cap and the junction with the stem (inflection point). The diameters of the short and long axes of the cap surface were measured at the longest and shortest cap lengths, respectively. The cap color was determined at three points in the middle of the cap using a colorimeter (Hangzhou CS-10, China) based on the CIE Lab system [15]. The color of the stipe was determined at three points in the middle part of the stipe.

### Development of markers based on SSR sequences

The SSR set was obtained from a previous study [16]. Primers were designed using Primer3 [17] with the following conditions: optimal primer length, 18–26 nucleotides; melting temperature, 45–55°C; product size, 100–350 bp; and G/C content, 45–55%. To distribute the markers evenly on the physical map, one SSR primer was manually selected from every 100 kb of the genome sequence of HMS012 and JHH021 [16] (S1 Table in S2 File).

Genomic DNA was extracted from lyophilized mycelia using GenEx Plant plus! Kit (GeneAll, Seoul, Korea). Polymorphic primers screened by preliminary PCR for HMS012 and JHH021 were used to genotype HMmp. PCR was performed using a thermal cycler PC 707–02 (ASTEC, Fukuoka, Japan) under the following conditions: denaturation at 95°C for 2 min, followed by 35 cycles of 95°C for 30 s, 52°C for 40 s, and 72°C for 30 s each; and a final extension at 72°C for 5 min. The total volume of the PCR reaction mixture was 10 μL containing 15 ng genomic DNA, 0.2 mM dNTPs, 0.25 U e-Taq DNA polymerase (SolGent, Korea), 1× buffer containing 2.5 mM Tris-HCl (pH 8.2) and 1.5 mM MgCl2, and 0.25 pM of each primer. Amplified PCR products were electrophoresed on a 1.5% agarose gel (Promega, Madison, WI) containing Safeview Classic (iNtRON Biotechnology, Sungnam, Korea). The SSR polymorphisms were scored based on the presence or absence of bands.

### Linkage map construction

Each marker was scored individually for the parents and mapping populations. Marker genotype data were compiled into a single spreadsheet. Linkage analysis of the markers, estimation of the recombination frequencies, and determination of the linear order of the loci were performed using JoinMap 4.0 [18]. Segregation patterns were assigned to each marker following the "HAP" data entry notation (<a>, <b>). Markers with highly distorted segregation ratios were filtered using the chi-square test (*P* < 0.001). Markers were placed into linkage groups (LGs) using the "logarithm of the odds (LOD) groupings" and "create groups for mapping" commands with the Kosambi map function [19]. The order of the markers in the linkage groups was established with a minimum LOD score of 5.0 (except for LG10 and LG11, LOD = 3) and a recombination fraction of 0.45. MapChart for Windows [20] was used to draw the LGs and QTLs.

### Statistical analyses

Quantile-quantile plots were used to test for normality and assess the distribution of values. The effect of genotype on each trait was determined using one-way analysis of variance (ANOVA). To identify transgressed segregation in the offspring, multiple comparisons were conducted among four genotypes using the Student-Newman-Keuls (SNK) test. The broad-sense heritability (*H*^*2*^) for traits was determined as follows: *H*^*2*^ = σG^*2*^ / *σP*^*2*^, where *σG*^*2*^ is the genetic variance, and *σP*^*2*^ is the phenotypic variance obtained by regression analysis. Correlation coefficients (CCs) for pairs of traits were obtained using Pearson’s procedure. The statistical significance of the differences between the means of Heuktar and Miso traits was determined using a two-sample t-test. Standard deviation and coefficient of variation (CV) were calculated for all 17 traits. Data analyses were performed using open-source software R [21].

### QTL analysis and molecular marker validation

The QTL analysis was performed using the composite interval mapping (CIM) program of Windows QTL Cartographer, version 2.5_011 [22]. The five most significant cofactors identified by forward and backward regressions were added as cofactors in the CIM step (Model 6) using a window size of 10 cM, five control markers, and a step size of 1 cM. LOD thresholds for significance were calculated using 1,000 permutations at a significance level of 0.05. QTLs above the significance threshold were considered significant, and the likelihood ratio (LR) test statistic was expressed as the LOD score (LOD = 0.2171 LR). The confidence interval (CI) was calculated from the points on the genetic map corresponding to a decrease in the LOD score by 1 (bar) and 2 (line) units from the highest peak. The approximate position of a QTL was considered the maximum LOD peak. The percentage of phenotypic variance and individual additive effects explained by a single QTL were calculated as *R*^*2*^ values at the highest probability peaks. *tR*^*2*^ was obtained from the Windows QTL Cartographer. The QTLs were named as follows (Y/NoS/E/P/L/LoS/T/SoC/Ct/DI/Ds/Lc/ac/bc/Ls/as/bs)x_y, where Y is the yield, NoS is the number of valid stipes, E is the earliness, P is the number of days required for pinheading, L is the entire length of the fruiting body, LoS is the length of the stipe, T is the thickness of the stipe, SoC is the shape of the cap, Ct is the thickness of the cap, DI and Ds are the diameters of the cap on the long and short axes, Lc, ac and bc are the colors of the cap [15], Ls, as and bs are the colors of the stipe, x is the LG, and y is the LOD value.

The QTL markers identified in this study were validated in the inbred lines. A second segregating population (HMmp2) comprising 110 monokaryons was constructed for genotyping. The second dikaryotic population (SGHMmp2) was prepared for phenotyping by mating HMmp2 with the tester. The closest SSR markers linked to the QTL of the traits were used to amplify the polymorphic regions between alleles. The enhancement rate of the selection precision was calculated by comparing the average phenotypic values between populations grouped by PCR-amplified polymorphic bands. Count selection was performed if a marker had a negative additive effect.

### Gene prediction of QTLs and transcript expression level analysis

Draft genomic sequences of HMS012 and JHH021 [16] were used to predict the candidate genes mainly responsible for cap color, number of stipes, yield, and cap shape. A sequence of approximately 1,000 kb, corresponding to the region around the closest marker (± 500 kb), was then analyzed using the FGENESH (http://www.softberry.com/), EMBL-EBI (http://www.ebi.ac.uk/), PROSITE (http://www.expasy.ch/sprot/prosite.html) and UniProt (http://www.uniprot.org/) programs to predict the genes present and their functions. The results were deposited in *P*. *ostreatus* genome browsers (http://web.seeders.co.kr/hms/ and http://web.seeders.co.kr/jhh/).

Transcript levels of the five candidate genes (glutathione S-transferase-like proteins: ustS (GST), glutathione disulfide reductase (GSR), cystathionine beta-synthase (CBS), hydroxyacylglutathione hydrolase (HAGH), and MYB transcription factor) for cap yellowness were measured using qRT-PCR. In the second segregated population (HMmp2), three individuals with high and low levels of cap yellowness were selected. Total RNA was extracted from 50 mg of homogenized mycelia using the TRIzol reagent (Invitrogen, Carlsbad, CA, USA). The isolated RNA was transcribed into cDNA using a PrimeScript™ II 1st strand cDNA Synthesis Kit (Takara, Japan) according to the supplier’s protocols. cDNAs from each of the three hybrids with high- and low-cap yellowness were pooled in equal amounts to produce two samples (high- and low-cap yellowness). The quantity and quality of the extracted RNA and cDNA were analyzed using a DS-11FX spectrophotometer (DeNovix, Wilmington, NC, USA).

qRT-PCR was performed on the two pooled samples of high- and low-cap yellowness to quantify the expression of candidate genes (GST, GSR, CBS, HAGH, and MYB transcription factor). The primer pairs used for qRT-PCR amplification of the internal fragments of these genes were designed using Primer3 and are listed in S2 Table in S2 File. Synthesized cDNA (100 ng/μL) was used as a template for qRT-PCR. qRT-PCR was performed as a duplex, where one duplex partner was used as an internal standard gene (*P*. *ostreatus* beta-tubulin rRNA), and the other duplex partner was one of the GST genes. The QuantStudio™ 1 Real-Time PCR System (Applied Biosystems, Carlsbad, CA, USA) was used for the reaction as follows: an initial step at 95°C for 30 s, followed by 40 cycles at 95°C for 3 s and 60°C for 20 s. One microliter of cDNA (100 ng/μL) was amplified in a 20.0 μL reaction using TB Green® Fast qPCR Mix (Takara, Japan) with each primer at a final concentration of 500 nM/L. Data analysis was performed using in-house QuantStudio™ software.

## Results

### Phenotypic variation of the SGHMmp and parental strains

The distribution of traits in the F1 population derived from the cross between Heuktari and Miso revealed that the key phenotypes (yield and cap lightness) of the HMS012 x JHH021 hybrid fell within the intermediate range of the distribution (S2 Fig in S1 File). The SGHMmp phenotypes showed continuous variation with a near-normal distribution for almost all traits, as evidenced by the Q-Q plots (S3 Fig in S1 File); thus, no data transformation was required. Not all the SGHMmp traits had intermediate parental values (Table 1 and S3 Table in S2 File). The yield of the SGHMmp was 96.64 g, with a range of 50.0–176.8 g, which was unexpectedly lower than that of its parents, Heuktari and Miso. This trend was observed in length and stipe thickness, whereas the mean color traits declined between those of the parents (Table 1 and S3 Table in S2 File). Multiple comparisons (Student-Newman-Keuls) test (*P* < 0.05) revealed six traits exhibiting transgressive segregation in both directions (S3 Table in S2 File). For yield, four strains had higher values than HMS012 × JHH021 (F1), while eleven had lower. And for length, nine strains exceeded the F1, while two fell below. Specifically, stipe thickness and cap color showed one-sided transgressive segregation. For stipe thickness, fourteen strains transgressed with higher values, while none transgressed with lower values. In terms of lightness, one strain transgressed with higher lightness, while twenty transgressed with lower lightness. Finally, for both redness and yellowness, eighty-six strains transgressed with higher values, while none transgressed with lower.

**Table 1:** Phenotypic characteristics and broad-sense heritability of the segregating population crossed with the tester.

Trait (unit)	Code	Mean (SD)	[min–max]	CV (%)	*H* ^*2*^
**Yield-related traits**					
Yield (g)	Y	96.64 (25.63)	[50.0–176.8]	26.5	0.74
Number of valid stipes (each)	NoS	29.28 (9.13)	[10.8–59.3]	36.2	-
Period of pinheading (day)	P	4. 70 (0.99)	[4.0–9.0]	21.0	-
Earliness (day)	E	9.69 (1.08)	[8.0–14.0]	11.1	-
**Morphology-related traits**					
Length (mm)	L	77.59 (12.33)	[53.1–116.7]	16.7	0.72
Length of stipe (mm)	LoS	58.38 (12.93)	[27.4–98.1]	22.1	0.77
Stipe thickness (mm)	T	9.33 (1.77)	[6.6–16.8]	19.0	0.75
Shape of cap	SoC	2.30 (0.77)	[1.0–4.0]	32.5	-
Cap thickness (mm)	Ct	11.21 (1.87)	[6.3–16.3]	16.6	0.64
Cap diameter_long (mm)	Dl	30.48 (1.92)	[26.6–35.7]	6.3	0.27
Cap diameter_short (mm)	Ds	27.36 (1.78)	[24.2–28.65]	6.5	0.24
Cap lightness	Lc	47.80 (7.88)	[31.5–66.3]	40.1	0.74
Cap redness	ac	4.06 (2.78)	[-0.9–6.6]	26.8	0.81
Cap yellowness	bc	10.40 (0.07)	[3.5–16.0]	5.1	0.82
Stipe lightness	Ls	81.65 (4.22)	[67.4–91.3]	5.1	0.70
Stipe redness	as	2.99 (0.41)	[2.1–4.2]	13.7	0.31
Stipe yellowness	bs	6.27 (2.05)	[0.9–10.9]	32.6	0.63

Statistical analysis revealed CVs ranging from 5.1 to 40.1, depending on the trait (Table 1). The CVs of cap lightness and the number of valid stipes were 40.1 and 36.2, respectively. However, the cap yellowness and stipe lightness CVs were very low (5.1).

Eight of the seventeen traits (yield, length, stipe length, stipe thickness, cap lightness, cap redness, cap yellowness, and stipe lightness) showed broad-sense heritability (*H^2^*) values of 0.70 or higher (Table 1). These three traits had very low *H^2^* values (cap diameter_long, cap diameter_short, and stipe redness). The stipe number, earliness, pinheading period, and cap shape could not be calculated owing to insufficient data.

Stipe length, length, and yield showed relatively high CCs with other traits (S4 Table in S2 File). The yield showed CCs of 0.71 and 0.77 with stipe length and length, respectively. Cap thickness and stipe length showed a CC of -0.61 (*P* < 0.05). Stipe lightness was slightly correlated with cap lightness (*r* = 0.44) but strongly negatively correlated with stipe yellowness (*r* = -0.89).

### Genetic map of the HMmp (HMS012 × JHH021 F1)

**Fig 2:**
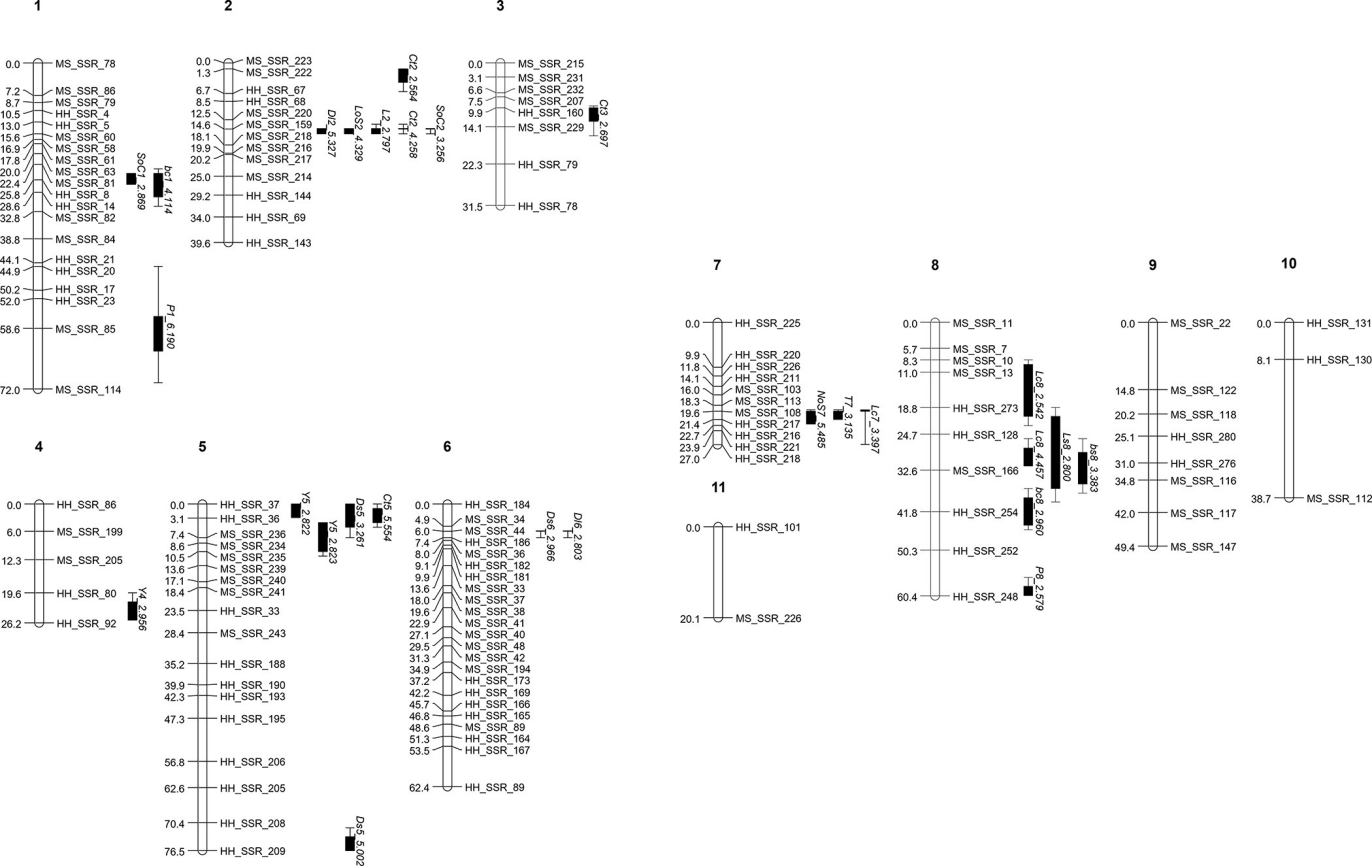
Location of QTLs responsible for traits on the *Pleurotus ostreatus* linkage map with LOD score. The genetic distance of markers in cM (Kosambi units) is indicated on the left side. The QTL nomenclature is described in the materials and methods section. The confidence interval is denoted by a bar (> LOD-1) and a line (> LOD-2) on the genetic map.

In total, 546 primer pairs were designed from a previous study [16], of which 265 (49%) amplified polymorphic fragments from 103 of the 110 progenies of the HMmp. Markers with highly distorted segregation (*P* < 0.001 by *χ2* test) were excluded from linkage analysis (*n* = 108). The 121 markers were successfully assigned to 11 linkage groups (LGs) with 2–23 markers in each LG. The LOD thresholds for LGs were higher than 5, with the exception of LG10 and LG11, which were > 3.0. LGs sizes ranged from 20.13 cM to 76.51 cM, totaling 503.71 cM for all LGs (average interval: 7.4 cM) (Fig 2 and S5 Table in S2 File). The ratio of physical to genetic distance was 80.5 kb/cM based on the previously reported size of the genome (40.55 Mbp) of *P*. *ostreatus*, Miso and Heuktari [16].

### QTL identification

QTLs for all analyzed traits were identified using CIM by a QTL Win-cartographer. QTLs with LOD thresholds of 2.5 (deduced by 1,000 permutation tests) or higher were therefore considered significant (Table 2). Twenty-seven QTLs were found for 14 of the 17 traits (no QTL for earliness and redness) distributed over LG1-8. Numerous QTLs were co-located in close genetic proximity to LG2, LG5, LG7, and LG8. On LG2 (14.51–14.63 cM), QTLs for length, stipe length, cap shape, cap thickness, and cap diameter (length) were located. Similarly, QTLs for yield, cap thickness, and cap diameter (short) were located on LG5 (1.01–5.09 cM). LG7 (19.27–22.40 cM) harbored QTLs controlling number of valid stipes, stipe thickness, and cap lightness. In contrast, LG8 (20.80–31.73 cM) was associated with stipe-related traits (lightness and yellowness), along with cap lightness.

**Table 2:** QTLs for yield and morphological traits found in the fruiting body of SGHMmp.

Trait	QTL	LG	CI (cM) ^a^	LOD ^b^	Nearest marker ^c^	Position (cM) ^d^	*R*^*2*^ (%)	*tR*^*2*^ (%)	Additive effect
**Yield-related traits**									
Yield	Y	4	21.60–25.60	2.956	HH_SSR_92	25.60	10.6	27.3	-8.592
Yield	Y	5	0.01–3.01	2.822	HH_SSR_37	1.01	10.8	27.4	-8.612
Yield	Y	5	4.09–10.54	2.823	HH_SSR_36	5.09	12.0	28.6	-8.952
Number of valid stipes	NoS	7	19.63–22.40	5.485	HH_SSR_217	20.63	22.0	27.7	4.556
Period of pinheading	P	1	56.04–63.60	6.190	MS_SSR_85	58.60	20.2	37.0	0.459
Period of pinheading	P	8	58.32–60.32	2.579	HH_SSR_248	60.32	14.5	31.3	0.388
**Morphology-related traits**									
Length	L	2	14.51–15.63	2.797	MS_SSR_159	14.63	8.7	35.2	-3.857
Length of stipe	LoS	2	14.51–15.63	4.329	MS_SSR_159	14.63	13.6	37.2	-4.907
Stipe thickness	T	7	19.63–21.4	3.135	HH_SSR_217	21.40	10.0	34.1	-0.605
Shape of cap	SoC	1	24.36–26.83	2.869	HH_SSR_8	25.83	9.6	30.3	0.248
Shape of cap	SoC	2	14.51–14.63	3.256	MS_SSR_159	14.63	11.0	30.4	0.362
Cap thickness	Ct	2	1.33–4.33	2.564	MS_SSR_222	1.33	7.8	36.5	0.535
Cap thickness	Ct	2	14.51–14.63	4.258	MS_SSR_159	14.63	12.4	41.1	0.660
Cap thickness	Ct	3	9.89–12.89	2.697	HH_SSR_160	11.89	13.1	49.5	-0.797
Cap thickness	Ct	5	1.01–4.09	5.554	HH_SSR_36	3.01	16.7	41.2	1.060
Cap diameter_long	Dl	2	14.51–15.63	5.327	MS_SSR_159	14.63	18.6	31.2	0.888
Cap diameter_long	Dl	6	5.90–5.97	2.803	MS_SSR_44	5.97	9.3	31.2	-0.659
Cap diameter_short	Ds	5	0.01–5.09	3.261	HH_SSR_37	2.01	10.4	36.3	0.595
Cap diameter_short	Ds	5	73.45–76.45	5.002	HH_SSR_209	76.45	16.2	35.9	0.779
Cap diameter_short	Ds	6	5.9–5.97	2.966	MS_SSR_44	5.97	9.3	31.2	-0.659
Cap lightness	Lc	7	19.27–19.63	3.397	MS_SSR_108	19.63	9.7	40.9	2.650
Cap lightness	Lc	8	9.34–20.80	2.542	HH_SSR_273	17.00	8.4	42.8	2.601
Cap lightness	Lc	8	27.73–31.73	4.457	MS_SSR_166	30.73	18.0	36.0	3.445
Cap yellowness	bc	1	24.36–29.62	4.114	HH_SSR_8	26.83	13.5	44.6	1.055
Cap yellowness	bc	8	38.65–44.78	2.960	HH_SSR_254	41.78	9.9	30.2	0.908
Stipe lightness	Ls	8	20.80–36.65	2.800	MS_SSR_166	32.65	8.7	35.1	1.282
Stipe yellowness	bs	8	28.73–35.65	3.383	MS_SSR_166	32.65	11.9	27.3	-0.724

a 1-LOD Support Interval. b LOD value at the LOD peak. c Nearest marker of the LOD peak. d LOD peak positions in cM.

### QTLs for yield-related traits

Three yield QTLs were discovered (one on LG4 and two on LG5), explaining 10.6%, 10.8%, and 12.0% of the phenotypic variation. The CIs of the QTLs was 3.00–6.45 cM. All additive effects on yield were negative, from -8.592 g to -8.952 g. For the number of valid stipes, *NoS7_5*.*485* was identified on LG7, with an additive effect of 4.556 each and *R*^*2*^ value of 22.0%. The CIs of this QTL overlapped those of *Lc7_3*.*397* and *T7_3*.*135*.

The pinheading period had two QTLs located on LG1 and LG8, with LODs of 6.190 and 2.579, respectively. Two QTLs explained 20.2% and 14.5% of the phenotypic variation. The former had the second-highest *R*^*2*^ of all traits. The additive effect of *P1_6*.*190* was 0.459 days, accounting for 9.77% of the mean of the period of pinheading. No significant QTLs for earliness were detected.

### QTLs for morphological characters

Five QTLs were detected for cap color traits, three for cap lightness, and two for cap yellowness. *Lc8_4*.*457* explained 18.0% of the cap lightness variation with an additive effect of 3.445. Two QTLs for cap yellowness were identified, with *bc1_4*.*114* explaining 13.5% of the phenotype. In addition, two QTLs for stipe lightness and yellowness were detected, with LODs of 2.800 and 3.383, respectively. Many QTLs for both cap and stipe color were located within a narrow region on LG8 from 9.34 to 44.78 cM. All color-related QTLs had positive additive effects except for stipe yellowness. The average CI of cap lightness and yellowness were 5.27 and 5.70 cM, respectively, and the average CIs of stipe lightness and yellowness were 15.85 and 6.97 cM, respectively. No significant QTL for cap or stipe redness was detected, possibly due to their low LOD values.

The QTLs for length and stipe length were represented on LG2 with identical CIs. In addition, these traits were strongly correlated (*R*^*2*^, 0.965), but their LODs (2.797 vs. 4.329) and variance explained (8.7% vs. 13.6%) were very different. The additive effects of the two QTLs were -3.857 (*L2_2*.*797*) and -4.907 mm (*LoS2_4*.*329)*, respectively. Stipe thickness was determined using a single QTL (LOD = 3.135) on LG7, with an *R*^*2*^ of 10.0%. This locus coincided with both cap lightness and the number of valid stipes (Fig 2).

Two QTLs for cap shape were identified on LG1 and LG2; they had LODs of 2.869 and 3.256 and variance explained of 9.6% and 11.0%. The CIs for cap shape on LG2 overlapped with those for cap thickness, length, stipe length, and cap diameter. The additive effects of these QTLs were 0.248 and 0.362, respectively. These were 10.8% and 15.7% of the mean value for this trait (2.3).

Four QTLs for cap thickness were identified, with the highest LOD of 5.554 and an *R*^*2*^ value of 16.7%, and were located on LG2, LG3, and LG5. All QTLs for cap thickness showed positive additive effects except for *Ct3_2*.*697*. The total variance explained (*tR*^*2*^) of *Ct3_2*.*697* was 49.5, which was the highest value among all QTL markers. Cap diameter_long and Cap diameter_short displayed five QTLs on LG2, LG5, and LG6, with LOD values ranging from 2.803 to 5.327. Specifically, the CIs of *Ds5_5*.*002*, *Ds6_2*.*966*, and *Dl6_2*.*803* were exclusively separated from the other trait QTLs, whereas *Dl2_5*.*327 and Ds5_3*.*261* overlapped with several trait QTLs. This explained variance by 9.3–18.6%.

### Selection efficiency with molecular markers

The enhancement rate of selection achieved using QTL markers varied among traits, ranging from 0.1% (length) to 50% (shape of cap) (S6 Table in S2 File) in the second dikaryotic population (SGHMmp2). Yield, the most important trait, was improved by 15.2% using markers HH_SSR_36, HH_SSR_37, and HH_SSR_92. The enhancement value of the combined markers was 12.1 g, which was lower than the sum of the enhancement values of the individual markers (19.3 g). Pinheading selection was improved by 13.4% using the combination of MS_SSR_85 and HH_SSR_248. The trait of cap thickness exhibited an improvement rate ranging from 2.1% to 30%, depending on the number of molecular markers utilized. The combination of HH_SSR_8 and HH_SSR_254 improved cap yellowness by 22.2%. However, length, length of stipe, cap diameter (short and long), cap lightness, and stipe colors showed low enhancement rates (below 10%) in selection with corresponding markers.

Overall, using multiple markers resulted in a higher selection precision than using a single marker. Specifically, the enhancement rate increased from 7.9% (single) to 11.7% (double) and 15.2% (triple) for yield, and similar trends were observed for other traits (S6 Table in S2 File). For traits carrying more than three markers, the combination affected the enhancement rate. In terms of yield, HH_SSR_37 and HH_SSR_92 improved by 9.4%, whereas HH_SSR_36 and HH_SSR_92 improved by 14.3%. This was also observed for the cap thickness and other parameters (S6 Table in S2 File). Notably, the selection precision for cap thickness varied among individual markers in the three marker combinations.

### Candidate genes of the color QTLs and comparison of RNA expression

**Fig 3:**
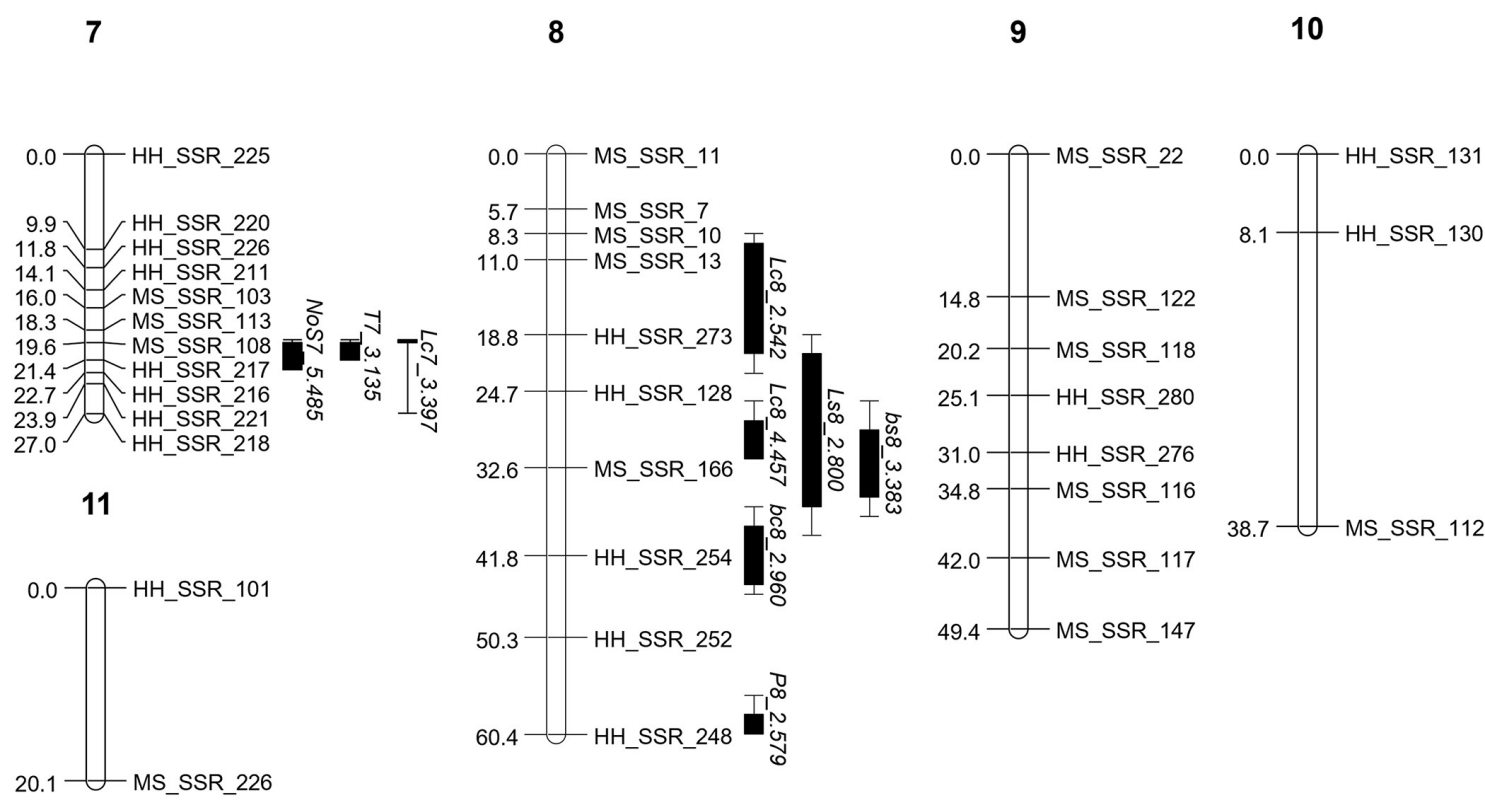
Transcript levels of candidate genes in high cap yellowness and low cap yellowness strains of *Pleurotus ostreatus*. Each set of three hybrids with high and low cap yellowness was selected from a second segregated population (HMmp2). Relative gene expression was estimated by the comparative 2−ΔΔCt method and was relative to the control using gene-specific primers. Gene expression was normalized to beta-tubulin expression and calibrated to the value for the high cap yellowness set, which was assigned a value of 1 (for A; B setting is the opposite), using the standard curve method (ABI). All assays were performed in triplicate. The error bars show the standard deviations for triplicate samples. The asterisk on the histogram indicates that the result is considered statistically significant (*, *P* < 0.05; **, *P* < 0.01; ***, *P* < 0.001). A, JHH00315-7 and JHH00323: Glutathione- S-transferase; B, JHH00313: glutathione disulfide reductase, HMS00363: Hydroxyacylglutathione hydrolase, HMS09261: Cystathionine beta-synthase, and JHH00337: MYB transcription factor.

A total of 127 genes were predicted by computational analysis of the flanking sequences of the nearest markers linked to cap color QTLs with high LOD. Candidate genes previously reported to be involved in cap and stipe color were identified. MYB transcription factor, cytochrome P450, WD40 repeat-like protein, cystathionine beta-synthase, and glutathione pathway enzymes were detected (S7 Table in S2 File). However, tyrosinase, the key enzyme involved in melanin biosynthesis, was not identified. Notably, the flanking region of *bc1_4*.*114* (closest primer: HH_SSR_8) contained many glutathione and cysteine pathway genes, including four glutathione S-transferase-like proteins: ustS (GST), glutathione disulfide reductase (GSR), cystathionine beta-synthase (CBS), and hydroxyacylglutathione hydrolase (HAGH). The expression levels of four GST genes (JHH315, JHH316, JHH 17, and JHH 23) in the two bulked samples grouped by cap yellowness were analyzed using qRT-PCR. The transcript levels of the GST genes were significantly higher (95.0–99.9% CI) in the low-cap yellowness samples (1.5–8.1 folds) than in the high-cap yellowness samples (Fig 3A). In particular, JHH315 and JHH316 showed the most significantly different expression patterns between the two bulk samples based on cap yellowness (*P*<0.001), the others showed same trend with early two genes but statistical significance was lower (*P*<0.01). The transcription rate of the CBS (HMS09261) was significantly higher in the high-cap yellowness strains (*P*<0.01) (Fig 3B). GSR (JHH00313) and MYB transcription factor (JHH00337) exhibited up-regulation in high-cap yellowness samples, although their expression levels did not significantly differ between the groups (Fig 3B). The expression of all genes mentioned thus far elevated cellular contents of cysteine or glutathione in the high cap yellowness group, which is consistent with the high cap yellowness trait and supporting the expectation that cysteine and glutathione are essential for high cap yellowness. HAGH (HMS00363) expression was slightly lower (1.8%) in high-cap yellowness strains than low-cap yellowness strains (Fig 3B).

## Discussion

The substantial number of oyster mushroom fruiting bodies and varying developmental stages within a single bottle pose a challenge in establishing statistical significance, even when selecting representative individuals for analysis. Although phenotyping repetitions were limited, the trait’s CV was relatively low (14.6 for yield and 8.3 for cap lightness). This was accomplished by harvesting twice daily and implementing a well-conditioned cultivation system with a "multispan panel house." Most traits in SGHMmp showed normal distribution, likely because of the parental strains, Heuktari and Miso, with the distinctiveness of yield and morphological alleles and traits controlled by QTLs.

Six traits exhibited transgressive segregation. Notably, the three color components displayed a one-sided pattern, which may have been influenced by the tester. This is likely due to KNR2247’s high redness and yellowness of the cap (Fig 1). The SSR primers were not evenly distributed on the genetic map, possibly because of the different recombination rates at different genome sites, despite the even selection of SSRs from the genomic sequence (1/100 kb). A significant degree of distorted inheritance was detected in 40.7% (108/265) of the markers, consistent with previous studies on *P*. *ostreatus* (14%) and *P*. *eryngii* (12.7% and 20.4%) [8, 12, 23]. This bias is likely caused by basidiospore germination and growth rates depending on the genotype [12, 23]. Even with careful selection, early and fast-growing monokaryons might be selected first even though careful selection.

The resulting map comprised 11 LGs covering 503.71 cM (S5 Table in S2 File and Fig 2). The number of LGs was similar to that reported for other *Pleurotus* species, which have 11–13 LGs, but the total cM was approximately half of the previous values, covering approximately 1,000 cM [8, 10, 12, 23]. This discrepancy could be attributed to the lower recombination rate in this *P*. *ostreatus* population, which is generally known to occur in basidiomycetes. Furthermore, the ’Miso’ was bred by a protoplast fusion of *P*. *ostreatus* and *P*. *florida*, with the latter being distinct from *P*. *ostreatus* [24]. Therefore, the homology between Miso and Heuktari is expected to be low. It also might to be the cause of the high distortion rate in the marker segregation. The average ratio of physical to genetic distance (80.5 kb/cM) was higher than that reported in previous studies, 35.1 kb/cM for *P*. *ostreatus* and 32.2 kb/cM for *P*. *eryngii* [12, 23]. These results could be attributed to the smaller number of markers used in this study (121) compared to *P*. *ostreatus* (189) and *P*. *eryngii* (256). A lower ratio of physical to genetic distance is better for estimating candidate genes adjacent to a QTL. While segregation distortion has little impact on QTL research [25] we thought that excluding the bias marker was a better approach because even a small genotypic error could significantly increase cM [26]. Eight traits had heritability over 0.7 in SGHMmp (Table 1), meaning the genotype contributed 70% of phenotypic variations of the traits. As a result, numerous traits were anticipated to have major QTLs with high LOD and *R*^*2*^ values. However, the QTLs identified in the present study do not support this assumption, which is inconsistent with previous studies [9, 12]. QTL detection is dependent on heritability [27] as well as other factors. CVs of each trait were relatively high (Table 1). A normal distribution with a high CV indicates high trait variability in the population. This can improve the accuracy of QTL detection [9] and lead to shorter CIs for QTLs [12], resulting in improved genotype discrimination and limited regions for candidate genes. This study identified twenty-seven QTLs for 14 out of the 17 traits. Similarly, Larraya et al. (2003) [9] reported twenty-eight QTLs for 13 traits of *P*. *otreatus*. The QTLs for cap color were found in *P*. *cornucopiae* [10]. This study led to the identification of the gene responsible for the cap color by RNAi [11]. In this study, the traits and cultivation methods differ from those in the previous reports. Various traits were assessed for the first time due to commercial evaluation, including yellowness and redness, cap shape and thickness, and the period of pinheading. A bottle cultivation system employing different media and harvest flushes was used in comparison to a bag cultivation system as well.

Several QTL-hotspots were identified across traits (Fig 2). Five and three QTLs for yield and morphology-related traits were identified on LG2 (14.51–14.63 cM) and LG5 (1.01–5.09 cM), respectively. LG7 (19.27–22.40 cM) harbored QTLs controlling the number of valid stipes, stipe thickness, and cap lightness. Additionally, QTLs affecting cap colors were observed on LG8 (20.80–31.73 cM). Studies in the genus *Pleurotus* have shown that fruiting body-QTLs were clustered in a narrow genetic region rather scattered across it. A QTL study with *P*. *ostreatus* identified one locus, L16875 on LG7, associated with five traits: clean fruiting body weight, fruiting body number, precocity, first-flush yield, and yield. LG4 also had 4 trait QTLs (yield, first-flush yield, fruiting body number, and fruiting body color) were located within a narrow genetic region [9]. In another QTL study with *P*. *eryngii*, LG1 (65.4–115.3 cM) harbored QTLs for yield, length, quality, earliness, period of pinheading, and cap color (lightness). LG7 (38–65 cM) also exhibited QTLs for traits very similar to those found on LG1, with the exception of cap color [12]. A locus on LG7 was identified as a QTL for yield-related traits in all three studies. Cap-related QTLs were also found in hotspot regions on LG5 in *P*. *eryngii* [12] and LG8 in this study. It suggests that these loci have pleiotropy or are independently linked [28, 29]. This conjecture is reinforced by the substantial correlation observed between the phenotypes of selected traits, including cap shape, diameter, and length (average CC of 0.465 for LG2). In addition, a significant correlation was observed between the lightness and yellowness levels of both the cap and stipe (LG8). The existence of numerous QTLs for yield, cap thickness, and cap diameter_short in the same chromosomal area concurred with their elevated CCs (LG5). However, determining whether these compounding QTLs stem from a pleiotropic impact or simply from a close biological outcome is difficult. Further detailed mapping is imperative to obtain a better understanding of these circumstances.

The QTL for pinheading showed a long CI, possibly because of the relatively short period of pinheading and ambiguous pinheading criteria in *P*. *ostreatus*. In particular, the average CIs of color QTLs was wider than that of other traits, 7.14 for colors vs. 2.57 for other traits (Table 2). This indicates that the phenotypic variation of colors (L, a, and b) of the cap and stipe were not substantial in the population. Additionally, rapid color changes during growth can blur trait distinctions between individuals before they are scored [11]. More frequent measurements are required than bi-daily measurements for color traits in *P*. *ostreatus*. The presence of two or three major loci related to cap color (lightness) is consistent with the results of previous studies [9, 10, 12]. The QTLs responsible for controlling cap color (lightness) were found on two LGs (7 and 8) in this study, similar to *P*. *ostreatus* (LG4 and 10) [9]. In *P*. *eryngii*, 12 QTLs associated with cap color (lightness) were located on three narrow regions (LG1, 5, and 10) with the highest *tR*^*2*^ value of 44.87% [12], and in *P*. *cornucopiae*, three major QTLs were detected on LGs 4, 5, and 7 [10]. One plausible explanation could be the limited precision of cap color assessment and marker number. The *R*^*2*^ values (proportion of phenotypic variance explained) of QTLs in *P*. *ostreatus* in this study were lower than those in *P*. *eryngii* [12] and similar to those in *P*. *cornucopiae* and *P*. *ostreatus* [9, 10]. The inconsistencies of LG name corresponding cap color QTLs between species might be expected due to the differences in genetic maps between species. Instead, the number of major QTLs and their *R*^*2*^ values are more important. The number of major QTLs associated with cap color (lightness) is estimated to be around three, which falls within the range of previous reports [9, 10, 12].

Four QTLs for cap thickness were identified, and this trait was reported to have a high correlation coefficient (0.866) with individual weight [30]. Two QTLs for cap diameter_long were identified, consistent with the previous report of *P*. *eryngii* [12]. The QTLs for cap thickness and cap-diameter were firstly analyzed in this study.

Selection accuracy by the application of QTL markers improved in the inbred lines (S6 Table in S2 File). Enhancement rates averaged a low 9.8%. The markers’ accuracy has been negatively impacted by the quantitative nature of the target traits. The enhancement rate of the cap shape was notably high, at 50%. This observation suggests the long-lasting nature of the cap shape during development. Although the markers did not significantly improve cap lightness, the *R*^*2*^ values of both cap shape and cap lightness were very similar. It was assumed that a higher *R*^*2*^ value (the proportion of phenotypic variance explained by the QTL) of the QTL would result in a corresponding increase in the rate of enhancement. However, the enhancement rate of marker selection was not proportional to the *R*^*2*^ of the markers. The CC between the *R*^*2*^ of the marker and the enhancement rate varied across traits, ranging from -0.095 (cap lightness) to 0.675 (cap thickness) (S6 Table in S2 File). This discrepancy may be attributed to the limited number of SSR markers and populations. Fine substitution mapping is required to address this issue. More precise markers need to be developed due to the limitations of the markers used in this study. Ultimately, the specification of genes corresponding to the traits of interest can result in gene-based markers with high confidence enhancement rates.

Color is particularly important for breeders because of its association with consumer preferences [13, 30]. The cap color of oyster mushrooms is controlled by differences in the amounts and relative proportions of eumelanin and pheomelanin [31]. Pheomelanin (yellow or red color) is biosynthesized from DOPA-quinone in the presence of cysteine or glutathione (sulfur-containing biomolecules) in a cell. Notably, the flanking sequence of *bc1_4*.*114*, which confers cap yellowness, encoded several glutathione and cysteine pathway genes (S7 Table in S2 File). GSR catalyzes the reduction of glutathione disulfide to the sulfhydryl form of GSH. CBS is a critical enzyme in the cysteine biosynthesis pathway [32]. HAGH catalyzes the synthesis of GSH from hydroxyacylglutathione. Those enzymes increase the level of glutathione or cysteine in the cell, whereas GST catalyzes conjugation reactions with GSH for detoxification, thereby decreasing the level of GSH. (S4 Fig in S1 File). The GST may play important roles in pheomelanin biosynthesis [33]. Hematopoietic prostaglandin D2 synthase is a superfamily of GST whose expression is downregulated in yellow plumage chickens.

As reported previously, GSR, CBS, and HAGH may show high expression, whereas GST may show low expression in high-cap yellowness strains (S4 Fig in S1 File). According to the qRT-PCR results, the strains with high-cap yellowness showed significantly higher transcription levels of the CBS gene (HMS09261). The GSR gene (JHH00313) also exhibited higher expression in these strains, although this difference did not reach statistical significance. In addition, all four GST genes (JHH00315-7) were transcribed at significantly higher levels in the low-cap yellowness samples than in the high-cap yellowness samples (Fig 3). However, the expression of the HAGH gene was unexpectedly down-regulated in high-cap yellowness samples.

The MYB transcription factor, Cytochrome P450, and WD40 repeat-like protein were also located in the vicinity of color QTLs. The MYB transcription factor regulated tyrosinase mediating the rate-limiting step in melanin biosynthesis (S4 Fig in S1 File) [34]. The MYB transcription factor has been identified as a regulator that controls various developmental processes in plants, and its role in governing pigment accumulation in fruit has been investigated [35]. The MYB transcription factor gene exhibited increased expression in high-cap yellowness strains. However, carotenoid biosynthesis has not been established in this species, thus, its role in the pigments remains unclear. Cytochrome P450 has been proposed as a potential causative gene for melanogenesis in *Hypsizygus marmoreus* [36].

Overall, these findings support that the levels of GSH and cysteine may be associated with either eumelanin (if low) or pheomelanin (if high) pigmentation. This is the first study of candidate genes for cap yellowness in *P*. *ostreatus*. However, definitive confirmation of these results will require further evidence from artificial gene expression manipulation, such as using CRISPR-Cas9 with strong candidate genes in the list of S7 Table in S2 File. Similarly, any other QTLs identified in this study may be suitable candidates for gene identification. These outcomes could be valuable in enhancing the comprehension of the genetic inheritance of agronomically important traits in *P*. *ostreatus*.

## Conclusions

We successfully constructed an 11 LGs genetic linkage map of *P*. *ostreatus* based on the segregation of SSR markers comprising 121 loci in “F2” monokaryons. Our study identified a total of 27 QTLs for 14 traits, explaining between 7.8% and 22.0% of the phenotypic variation. Several glutathione and cysteine pathway genes have been identified as candidate genes involved in cap yellowness. Some molecular markers, such as cap shape and cap thickness, can be easily employed for selection, even though in lines derived from *P*. *ostreatus* Heuktari and Miso. The QTLs identified in this study, along with candidate genes, could serve as strong probes to locate corresponding genes, leading to the development of more precise gene-based markers in the future.

## Supporting information

S1 File (ZIP)

S2 File (ZIP)
