# Correction: Quantitative trait loci analysis for molecular markers linked to agricultural traits of *Pleurotus ostreatus*

**DOI:** 10.1371/journal.pone.0329953

**Published:** 2025-08-06

**Authors:** Jae-San Ryu, Bokyung Park, Arend F. van Peer, Kyeong Sook Na, Song Hee Lee

Fig 3 is uploaded incorrectly. Please see the correct Fig 3 here.

**Fig 3:**
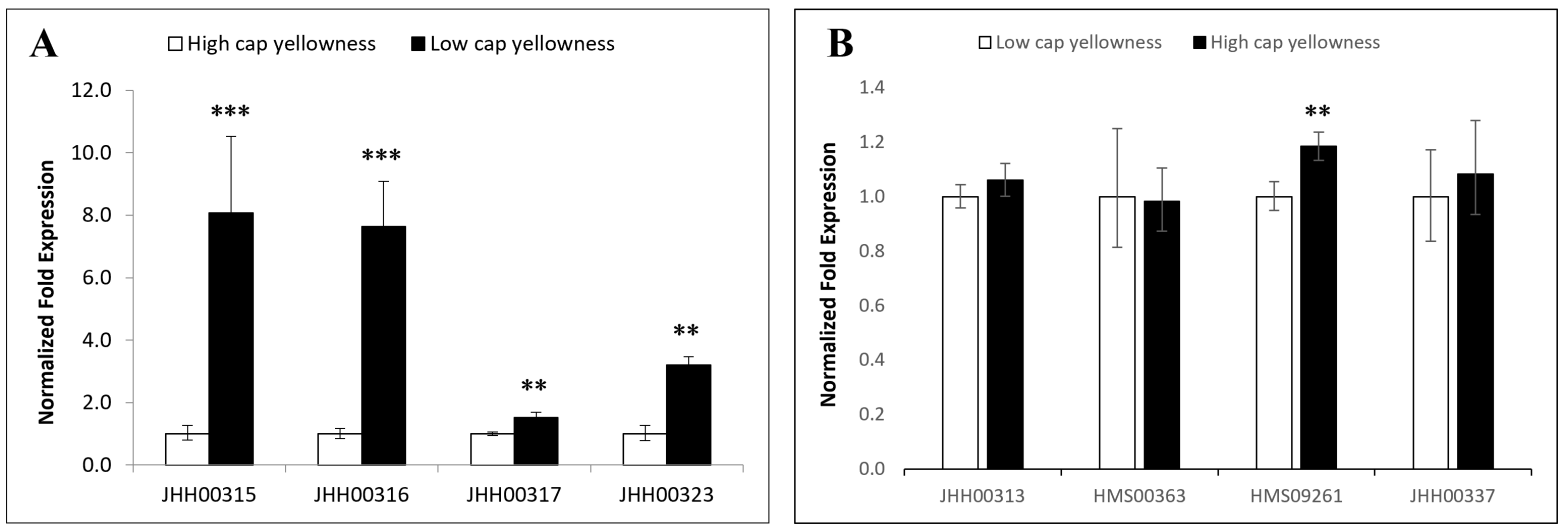
Transcript levels of candidate genes in high cap yellowness and low cap yellowness strains of *Pleurotus ostreatus.* Each set of three hybrids with high and low cap yellowness was selected from a second segregated population (HMmp2). Relative gene expression was estimated by the comparative 2 − ΔΔCt method and was relative to the control using gene-specific primers. Gene expression was normalized to beta-tubulin expression and calibrated to the value for the high cap yellowness set, which was assigned a value of 1 (for A; B setting is the opposite), using the standard curve method (ABI). All assays were performed in triplicate. The error bars show the standard deviations for triplicate samples. The asterisk on the histogram indicates that the result is considered statistically significant (*, *P* < 0.05; **, *P* < 0.01; ***, *P* < 0.001). A, JHH00315-7 and JHH00323: Glutathione- S-transferase; B, JHH00313: glutathione disulfide reductase, HMS00363: Hydroxyacylglutathione hydrolase, HMS09261: Cystathionine beta-synthase, and JHH00337: MYB transcription factor.
