# Supplementary material for: Quantitative trait loci analysis for molecular markers linked to agricultural traits of Pleurotus ostreatus
Source: PLoS One. 2024 Aug 12;19(8):e0308832. doi: 10.1371/journal.pone.0308832 (PMC11318876; doi:10.1371/journal.pone.0308832)
Supplement: S1 File — (ZIP) [file pone.0308832.s001.zip › S1. Fig.pdf]

**S1 Fig. Phenotypic characteristics of the Heuktari and Miso cultivars.** Parents *Pleurotus ostreatus* strains of a segregation population were cultivated in the bottle cultivation system.

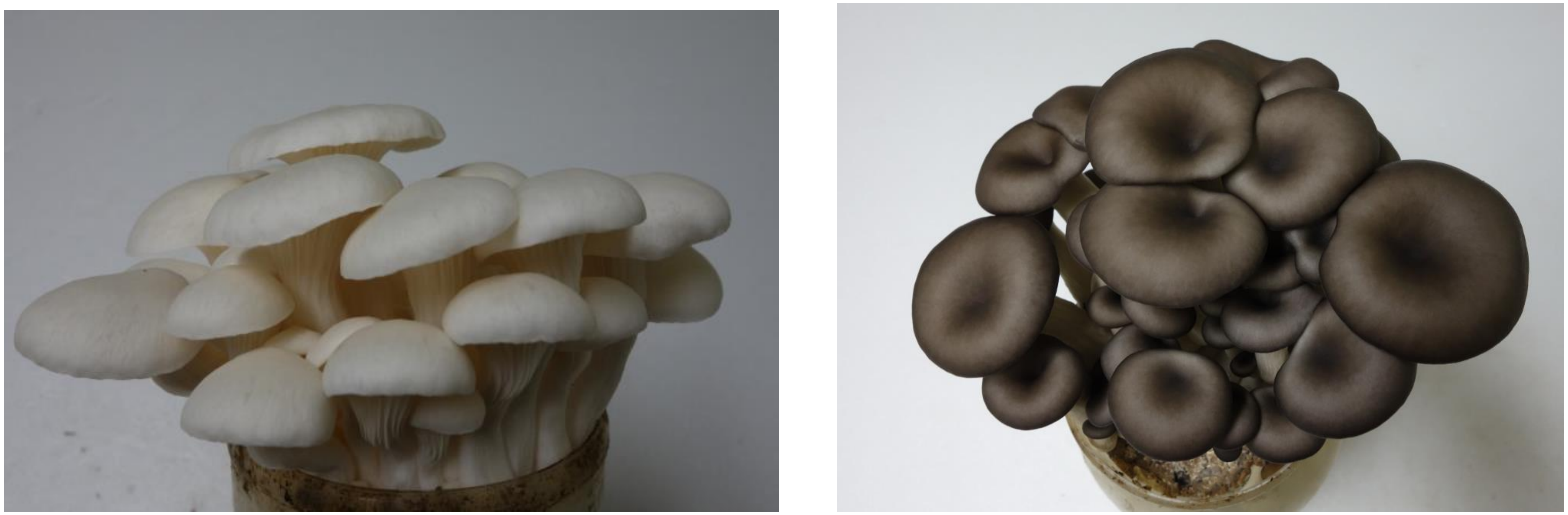

Miso Heuktari
