# Supplementary material for: Quantitative trait loci analysis for molecular markers linked to agricultural traits of Pleurotus ostreatus
Source: PLoS One. 2024 Aug 12;19(8):e0308832. doi: 10.1371/journal.pone.0308832 (PMC11318876; doi:10.1371/journal.pone.0308832)

S2 Fig. Distribution frequency of the F1 population from Heuktari x Miso for various traits. A total of 100 F1 individuals were hybrids between monokaryons from Heuktari and Miso, and they were evaluated after fruiting. The blue triangles indicate the values of HMS012 x JHH021.

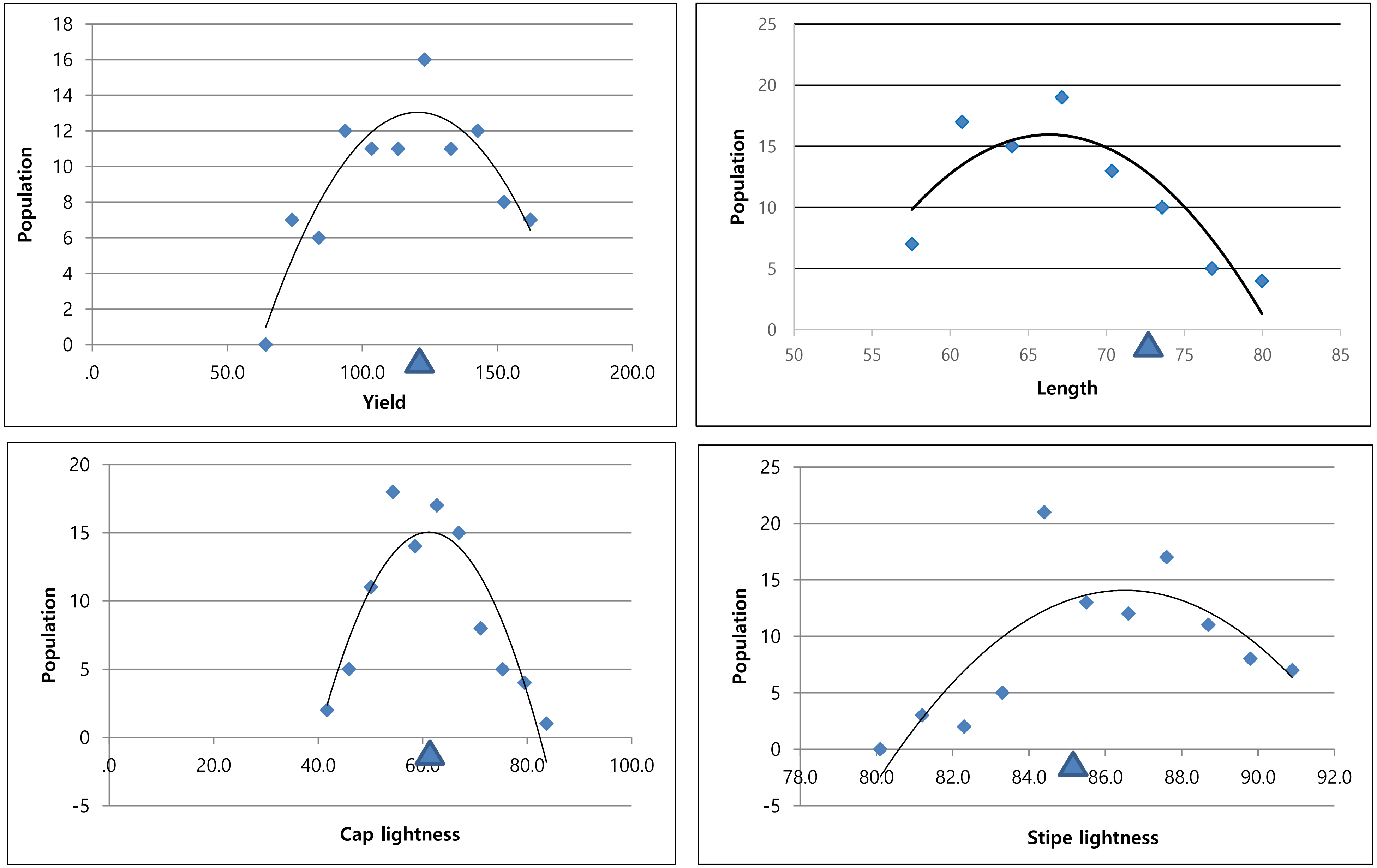

Supplement: S1 File — (ZIP) [file pone.0308832.s001.zip › S2 Fig.pdf]
