# Supplementary material for: Quantitative trait loci analysis for molecular markers linked to agricultural traits of Pleurotus ostreatus
Source: PLoS One. 2024 Aug 12;19(8):e0308832. doi: 10.1371/journal.pone.0308832 (PMC11318876; doi:10.1371/journal.pone.0308832)

S3 Fig. Q-Q plot of the traits in the second-generation hybrid population (SGHMmp).

Y:
Yield

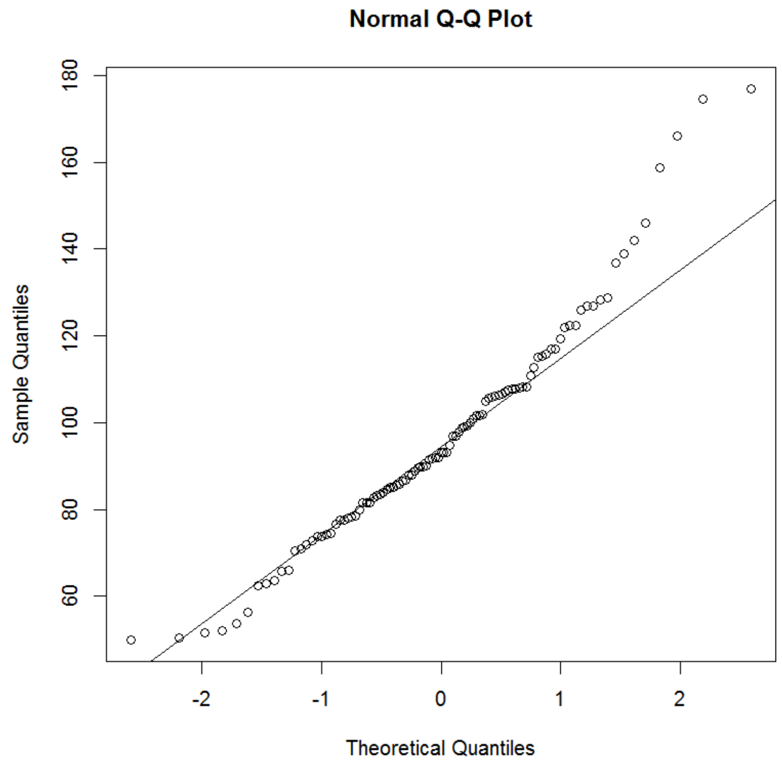

NoS:
Number of valid stipe

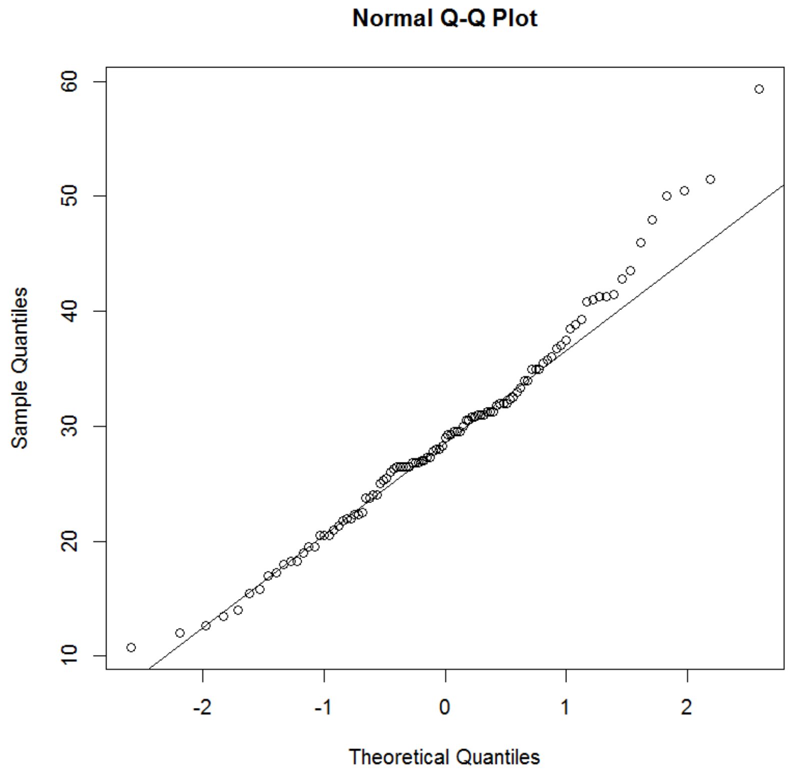

P:
Period of pinheading

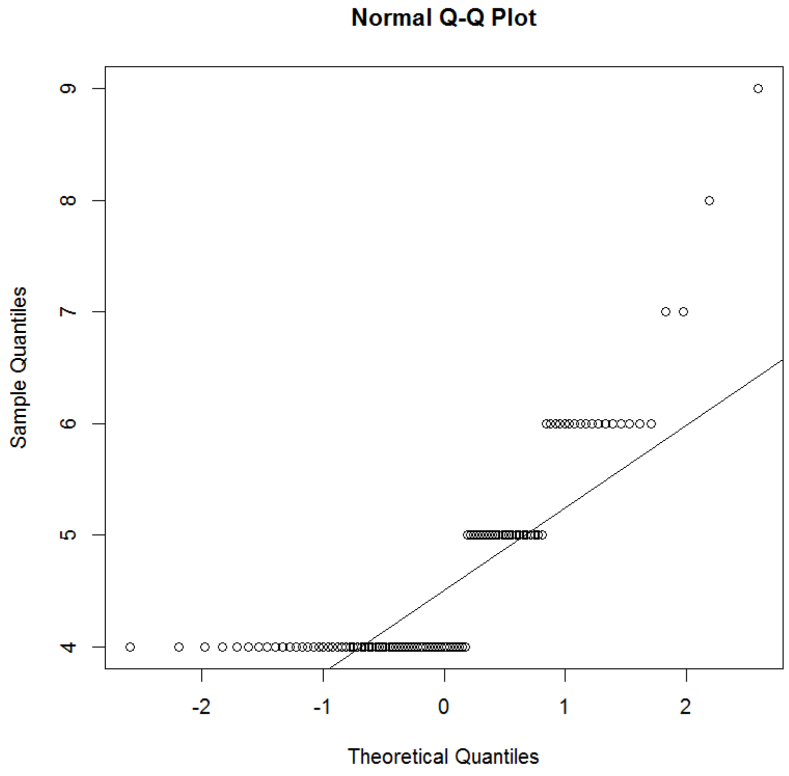

L:
Length

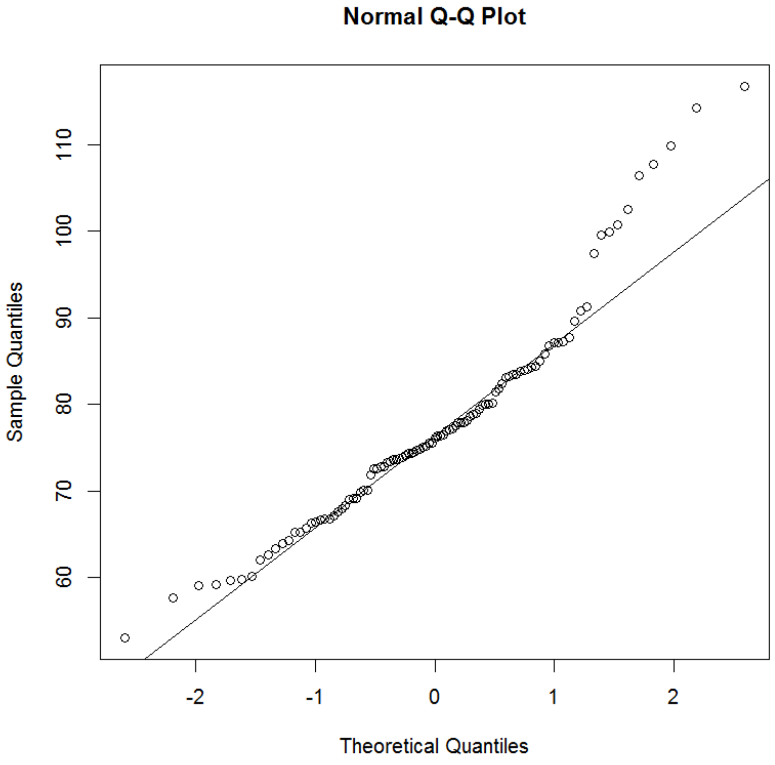

S3 Fig (continued)

LoS:
Length of stipe

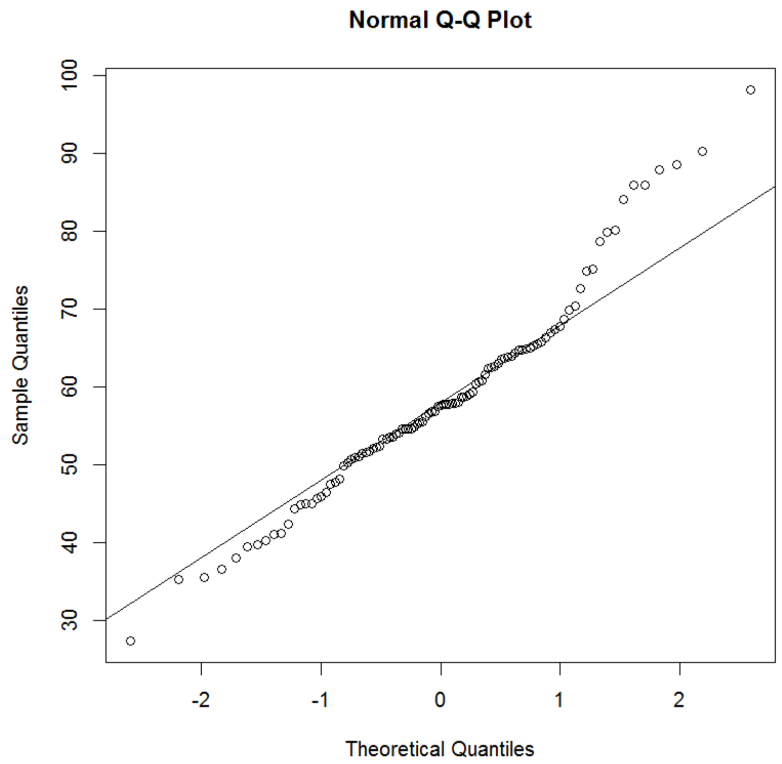

T:
Stipe thickness

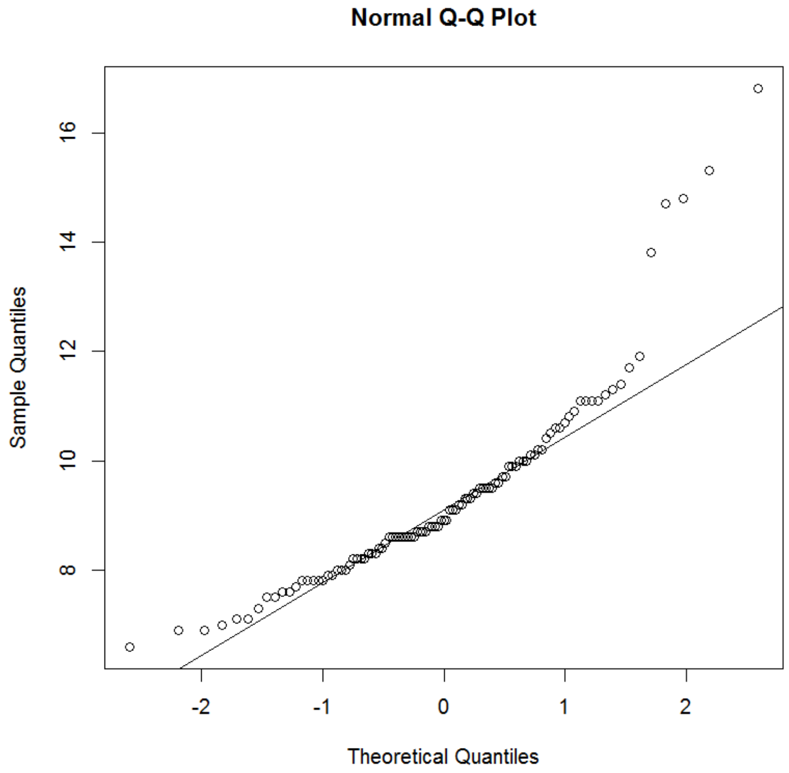

SoC:
Shape of cap

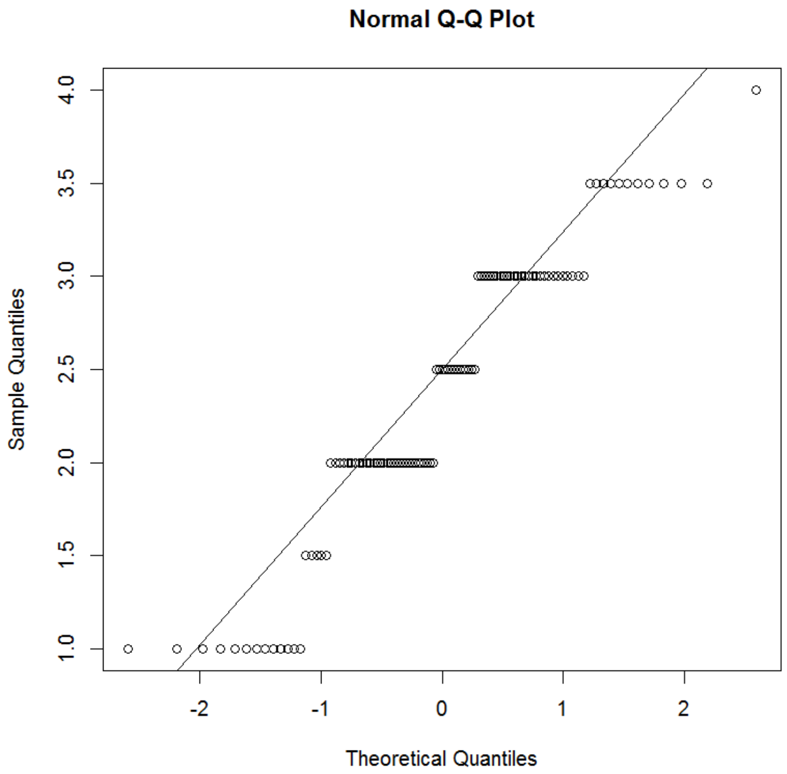

Ct:
Cap thickness

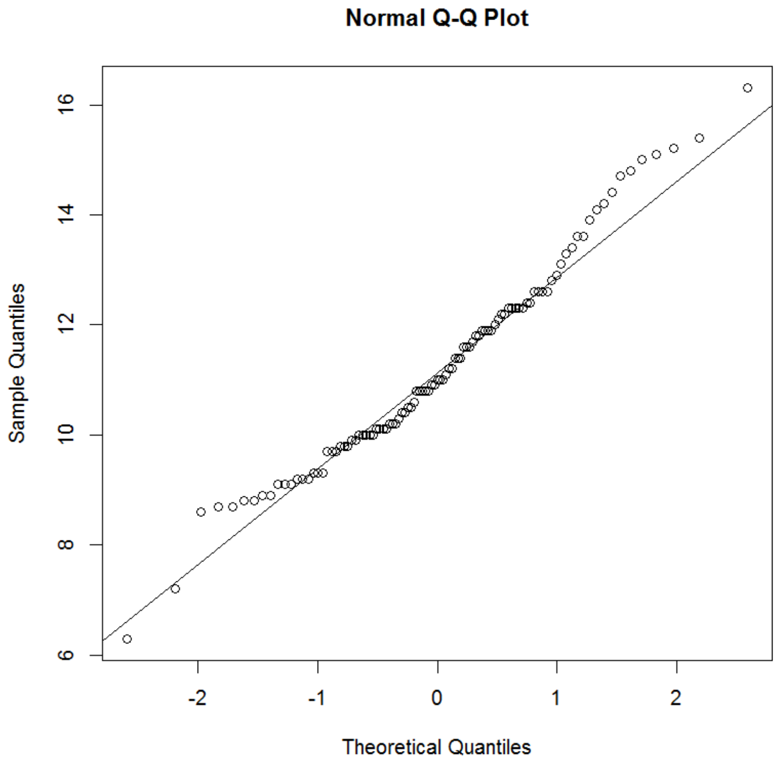

S3 Fig (continued)

Dl:
Diameter of long axis of cap

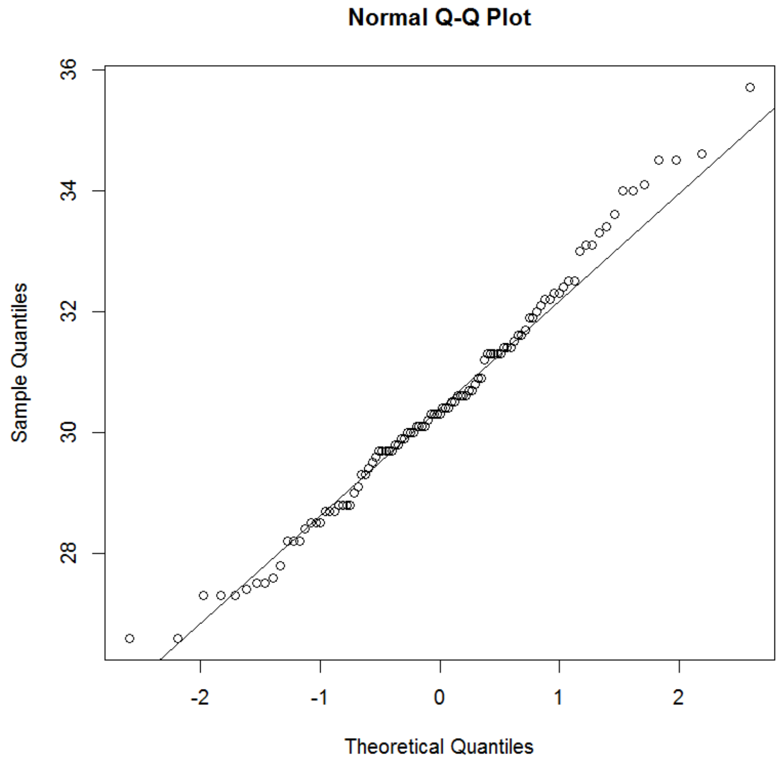

Ds:
Diameter of short axis of cap

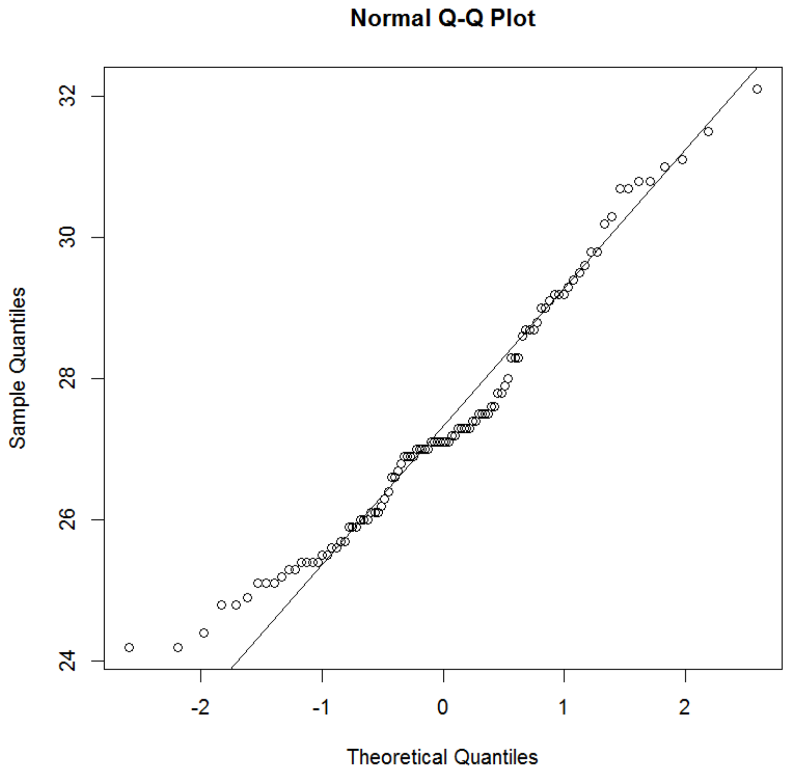

Lc:
Lightness of cap

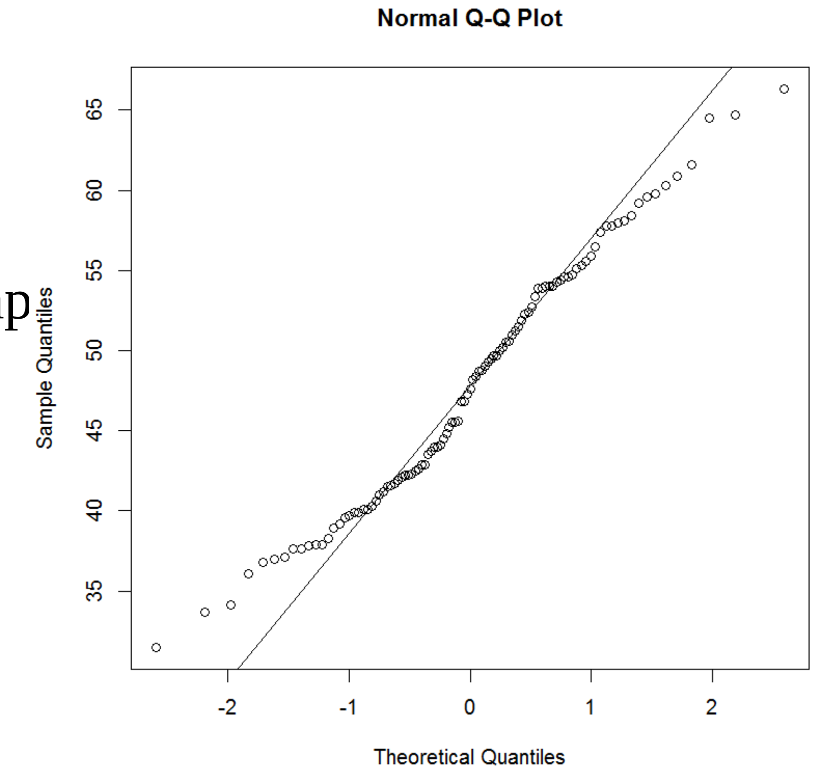

bc:
Yellowness of cap

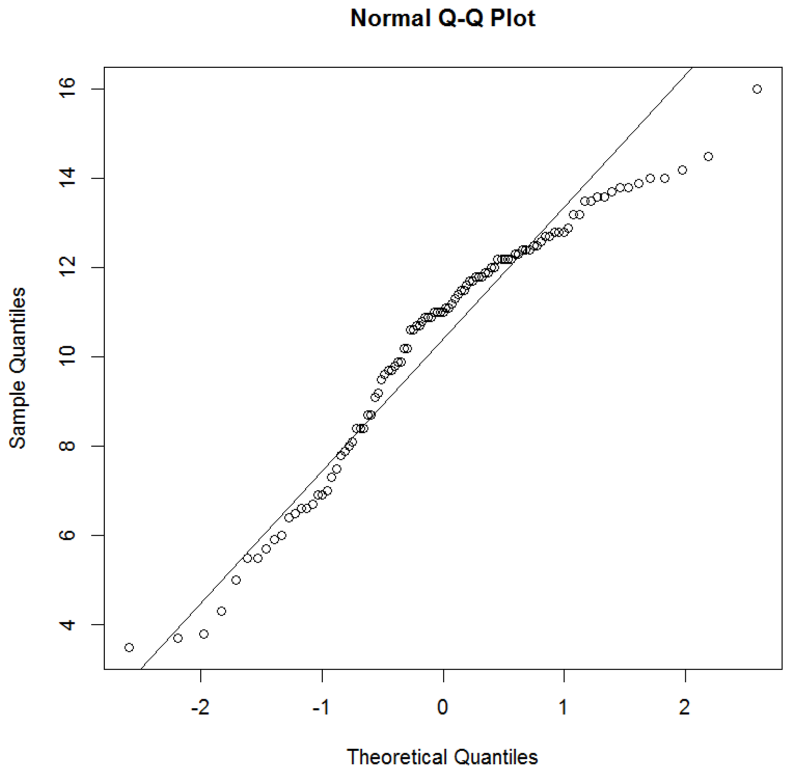

S3 Fig (continued)

Ls:
Lightness of stipe

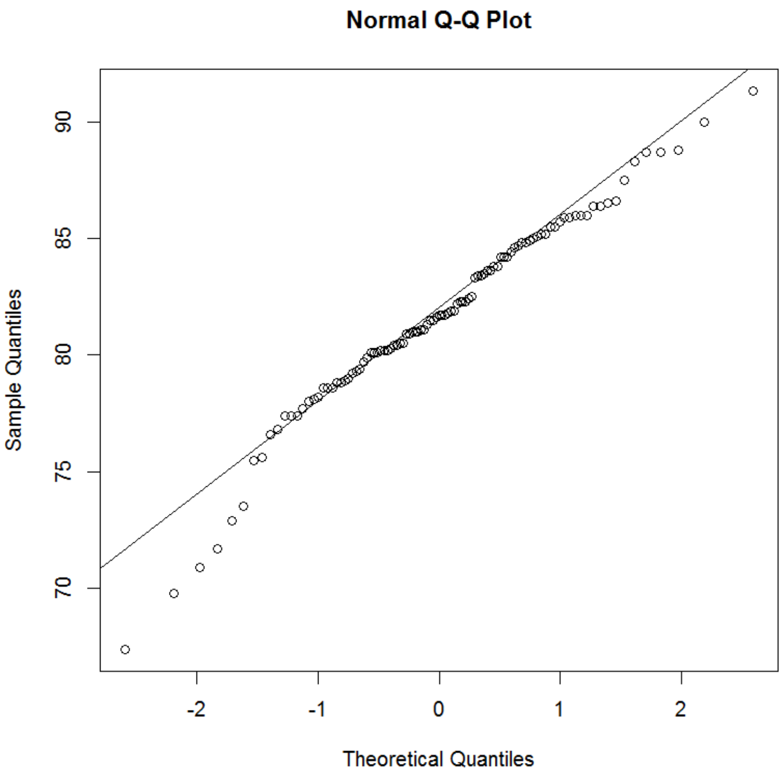

bc:
Yellowness of cap

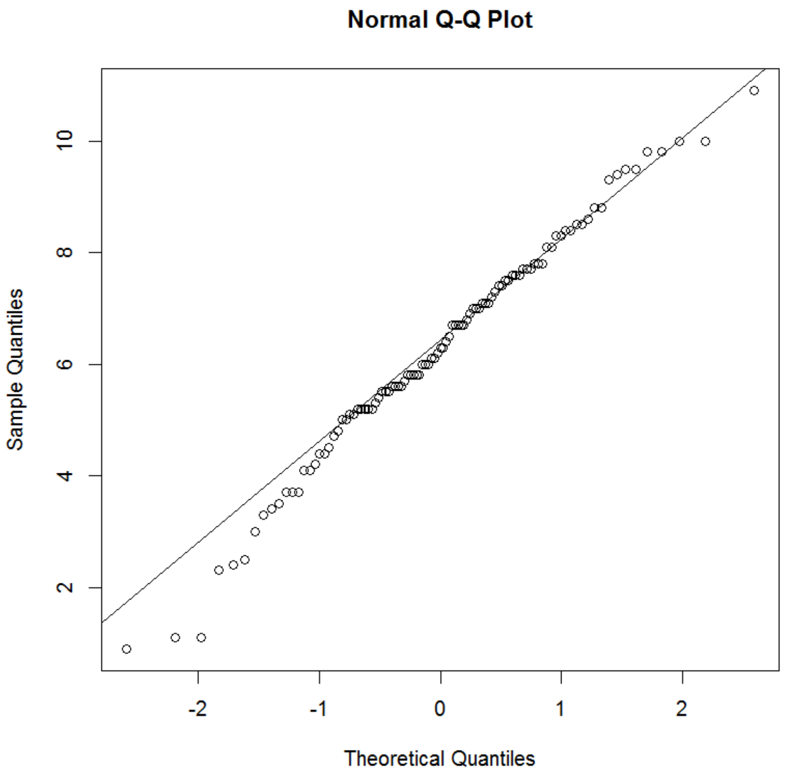

Supplement: S1 File — (ZIP) [file pone.0308832.s001.zip › S3 Fig.pdf]
