# Supplementary material for: Quantitative trait loci analysis for molecular markers linked to agricultural traits of Pleurotus ostreatus
Source: PLoS One. 2024 Aug 12;19(8):e0308832. doi: 10.1371/journal.pone.0308832 (PMC11318876; doi:10.1371/journal.pone.0308832)
Supplement: S1 File — (ZIP) [file pone.0308832.s001.zip › S4 Fig.pdf]

S4 Fig. Schematic model representing the Cysteine, Glutathione, and melanin biosynthesis, and their metabolism. Candidate genes and IDs related to cap yellowness are in bold. The dotted lines indicate that the materials within the yellow boxes participate in the DOPA-quinone → Cysteinyl-DOPA reaction. The boxed arrow indicates MYB-mediated regulation. Glu, glutamate; Cys, cysteine; Gly, glycine; γGCS, TYR, tyrosinase; γ-glutamylcysteine synthetase; GS, glutathione synthetase. DOPA, 3,4-dihydroxyphenylalanine; Cys-DOPA, Cysteinyl DOPA.

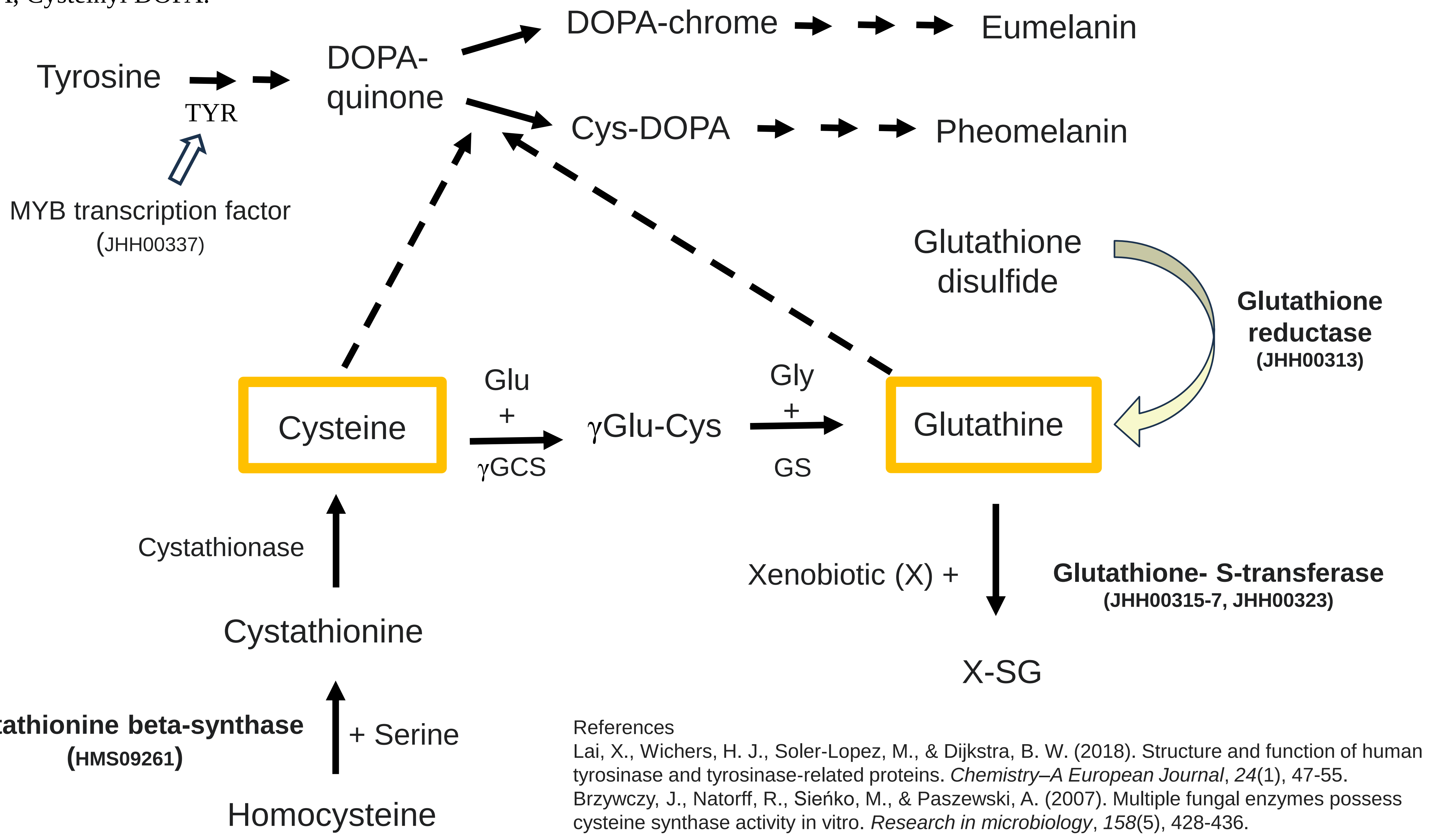

References
Lai, X., Wichers, H. J., Soler-Lopez, M., & Dijkstra, B. W. (2018). Structure and function of human tyrosinase and tyrosinase-related proteins. *Chemistry–A European Journal*, *24*(1), 47-55.
Brzywczy, J., Natorff, R., Sieńko, M., & Paszewski, A. (2007). Multiple fungal enzymes possess cysteine synthase activity in vitro. *Research in microbiology*, *158*(5), 428-436.
